# RegTransBase – a database of regulatory sequences and interactions based on literature: a resource for investigating transcriptional regulation in prokaryotes

**DOI:** 10.1186/1471-2164-14-213

**Published:** 2013-04-02

**Authors:** Michael J Cipriano, Pavel N Novichkov, Alexey E Kazakov, Dmitry A Rodionov, Adam P Arkin, Mikhail S Gelfand, Inna Dubchak

**Affiliations:** 1 Department of Microbiology, University of California Davis, Davis, CA, 95616, USA; 2 Lawrence Berkeley National Laboratory, Berkeley, CA, 94710, USA; 3 Sanford-Burnham Medical Research Institute, La Jolla, CA, 92037, USA; 4 A.A. Kharkevich Institute for Information Transmission Problems, Russian Academy of Sciences, Moscow, 127994, Russia; 5 Department of Bioengineering, University of California, Berkeley, CA, 94720, USA; 6 M.V. Lomonosov Moscow State University, Vorobyevy Gory 1073,, Moscow, 119991, Russia; 7 Department of Energy Joint Genome Institute, 2800 Mitchell Drive, Walnut Creek, CA, 94598, USA

**Keywords:** Transcriptional regulation, Prokaryotes, Comparative genomics

## Abstract

**Background:**

Due to the constantly growing number of sequenced microbial genomes, comparative genomics has been playing a major role in the investigation of regulatory interactions in bacteria. Regulon inference mostly remains a field of semi-manual examination since absence of a knowledgebase and informatics platform for automated and systematic investigation restricts opportunities for computational prediction. Additionally, confirming computationally inferred regulons by experimental data is critically important.

**Description:**

RegTransBase is an open-access platform with a user-friendly web interface publicly available at http://regtransbase.lbl.gov. It consists of two databases – a manually collected hierarchical regulatory interactions database based on more than 7000 scientific papers which can serve as a knowledgebase for verification of predictions, and a large set of curated by experts transcription factor binding sites used in regulon inference by a variety of tools. RegTransBase captures the knowledge from published scientific literature using controlled vocabularies and contains various types of experimental data, such as: the activation or repression of transcription by an identified direct regulator; determination of the transcriptional regulatory function of a protein (or RNA) directly binding to DNA or RNA; mapping of binding sites for a regulatory protein; characterization of regulatory mutations. Analysis of the data collected from literature resulted in the creation of Putative Regulons from Experimental Data that are also available in RegTransBase.

**Conclusions:**

RegTransBase is a powerful user-friendly platform for the investigation of regulation in prokaryotes. It uses a collection of validated regulatory sequences that can be easily extracted and used to infer regulatory interactions by comparative genomics techniques thus assisting researchers in the interpretation of transcriptional regulation data.

## Background

Activation and repression of gene expression in bacteria is usually mediated by DNA-binding transcription factors (TFs) that specifically recognize TF-binding sites (TFBSs) in upstream regions of target genes. Genes and operons directly co-regulated by the same TF are considered to belong to a regulon. Predicting the regulon of a transcription factor that binds DNA by detecting TFBSs in most cases requires the alignment of known binding sites to create a positional weight matrix (PWM). It is very important to filter out irrelevant sites and find TFBSs that are of higher confidence, and comparative genomics is the method of choice for this.

With the advent of new and cheaper sequencing technologies and ongoing sequencing projects such as GEBA [1], which aims to close the gaps in the bacterial tree of life, a lot of bacterial organisms are now being sequenced [2]. Of note is that not only are organisms with no close sequenced relatives being sequenced, but specifically groups of closely related organisms and multiple strains of the same species. This trend of sequencing can be successfully exploited when using comparative analyses, and already has been used in studying and predicting transcriptional regulation [3-6].

While many transcriptional regulation experiments are performed on model organisms, the existing experimental evidence can be transferred to other organisms by comparative methods. However, even closely related organisms can have different transcriptional regulation [7], thus prediction of binding sites and regulon inference in bacteria until recently has been mostly done by careful manual analysis [8-10]. Availability of experimental data on regulation for a wider range of organisms would be very helpful in automatic verification of computationally derived predictions of regulation. These verifications require well-designed databases accessible to prediction and analysis programs.

Eukaryotic transcriptional regulation data has been summarized in both commercial and open-source databases, such as TransFac [11], Pazar [12], and ORegAnno [13], widely used by the community. There are several gene regulation databases that focus on distinct microbial organisms such as *E. coli*[14,15], *B. subtilis*[16], Mycobacterium tuberculosis [17], and corynebacteria [18]. On the other hand, PRODORIC [19], PePPER [20] and SwissRegulon [21] cover a wide range of bacterial genomes.

RegTransBase, first introduced in 2007 [22], was built with the goal to cover a wide microbial diversity and provide a collection of curated experimental data to use in external computational tools. The current advanced version of RegTransBase: (i) contains a much larger set of manually collected experimental results (Table 1); (ii) has a brand new interface with novel capabilities for multi-level data navigation such as the new Classification Browser and new data aggregation tools such as the Putative Regulons Browser; (iii) is linked to associated analytical systems.

**Table 1:** Content of RegTransBase

		**2012**	**2006**
Articles Curated:		7213	2958
Experiments:		18877	7129
Organisms:			
	Phylum	11	9
	Genus	224	Not reported
	Species	666	128
Genes:		59754	7467
Sites:		13321	6470
Regulators:		1488	678
Effectors:		875	558
PWM		151	151
Putative Regulons	Total	6390	N/A
	Avg. Regulon/Genome	13	N/A
	Median Regulon/Genome	5	N/A

It is important to mention that we have recently developed two new resources – the RegPredict Web tool to support genomic reconstruction of transcriptional regulons in groups of closely related prokaryotic genomes [23], and the RegPrecise database to capture, visualize and analyze transcription factor regulons that were reconstructed [24]. We are working on the integration of RegTransBase, RegPredict and RegPrecise into a powerful platform for regulon reconstruction and analysis.

## Construction and content

### Experimental data annotation

The main objective during the article annotation phase for RegTransBase was to collect experimental evidences of transcriptional regulation and experimentally characterized TF binding sites. The main steps of the data collection. Described in detail in our previous article [22], are the following: search for relevant articles in PubMed [25], entry of data through a specialized annotator interface, quality control, mapping sites and genes to genomes, additional manual corrections (if necessary) and presentation of the data in the final format. The entry quality is controlled by a number of consistency and completeness checks. The genomic location of a specific feature (site or gene) is then recorded by the annotator as a signature (a DNA sequence fragment of sufficient length) that is then used to map all the features in the database to a wide range of the NCBI RefSeq genomes [26,27].

Each database entry describes a single experiment that is an experimentally determined relationship between several database elements. A single entry may describe an experiment and control, identical results obtained by different methods or the results of the application of one technique to several similar objects. Only original results are recorded, normally from the ‘Results’ or ‘Discussion’ sections of an article.

The types of experimental techniques form a controlled vocabulary. The following categories of experiments were accepted: (i) regulation of gene expression by a known regulator; (ii) demonstration that a gene encodes a regulatory protein (excluding proteins that do not directly bind DNA, e.g. protein kinases); (iii) experimental mapping of DNA binding sites for known regulators; (iv) identification of mutations in regulatory genes influencing expression of regulated genes; (v) computational prediction of binding sites.

The classes of elements in the database are: regulators (regulatory proteins and RNAs directly binding to DNA, with a well-defined binding site); effectors (molecules not binding DNA or physical effects such as stress, etc.); and positional elements. The latter are described as regions in DNA sequences. Positional elements form a hierarchy: locus > operon > transcript > gene and site; an elements may be a sub-elements of other elements of the same or higher levels (e.g., a site and a gene may be a sub-element of a operon).

All elements are linked to the corresponding experiments and together they are linked to the original article. As mentioned above, positional elements are mapped to genomes, thus if two independent articles describe regulation of the same gene, the data contained in these articles will be interlinked via this gene, but sites and other experimental data will be reported as independent entries.

Our original publication on RegTransBase [22] and the Help pages at http://regtransbase.lbl.gov provide more details on the procedure of experimental data annotation.

### Putative regulons from experimental data

The Putative Regulons section of RegTransBase provides a list of experimental sites along with a non-redundant list of target genes for each regulator. The process we undertook in developing this list of putative regulons from the manually curated data includes three steps.

First, we selected a subset of experiments using the following criteria: (i) the experiment describes a single regulator, (ii) a regulator and its regulated genes belong to the same genome, (iii) no computational predictions are included.

Second, from this subset we extracted the pairs ‘regulator-regulated gene’ for each genome, taking into account operon structure, that extend the list of regulated genes by adding other members of a particular operon. In some cases we see a particular pair of a regulator and an associated regulated gene in multiple entries in RegTransBase. We removed such redundant pairs from the list of regulator-regulated genes based on positional mapping.

Third, we compiled a list of putative regulons by unifying all ‘regulator-gene’ pairs with the same regulator.

### Manually curated position weight matrices

Each record in the Manually Curated PWM section of the database comprises a TFBS training set (alignment) created by an expert curator using published experimental data and manual in silico analyses. The curator first gathered information about a known transcription factor where a set of binding sites was known, created a summary of a description of this transcription factor by scanning published articles, and recorded its genomic location. The curator then annotated binding sites and their sequence, downstream gene, location in a published genome, and any published experimental evidence. In addition, curators supplied groups of organisms that they believe could be used when searching for homologous binding sites based on phylogenetic distance of organism and presence of a conserved transcription factor. Lastly, the curator recorded default scores and the expected distance a binding site would be from the start of a gene based on examination of the existing binding sites.

A PWM is automatically created in the RegTransBase database based on the TFBSs alignment. We then searched all recommended bacterial genomes using MAST [28]. We recorded all hits that passed the following criteria into the RegTransBase database: e-value of 1e-5 or better, it did not overlap coding regions and it was upstream of a predicted gene.

With each record, we provide the binding site location with a reference to a published sequence (usually NCBI RefSeq [26]), the sequence, the gene which is affected by the binding site, the evidence for the binding if any, any relevant articles pertaining to that site, and the transcription factor which binds the site. We also provide for download the sequence logo for the alignment, profiles and alignments in many different formats, and recommended options in using the profiles for searching other genomes (cut-off scores, distance from gene, taxonomy).

### Database statistics

As of November 2012, RegTransBase contains information on 666 bacterial species from 224 genera. This resource allows for access to the information on 19000 different experiments from about 7200 articles from as far back as 1977 until the present day (more details in Table 1).

## Utility and discussion

Our goal is to provide a comprehensive resource to the greater genomic community to allow for easy transfer of known binding site information as well as tools for discovering interesting regulatory interactions in groups of organisms. We believe that by using a comparative approach, new genomes could be more easily annotated, and this approach can help facilitate the discovery and expansion of regulons in a wide range of organisms.

### Database access and features

RegTransBase is freely accessible via a user-friendly web interface at http://regtransbase.lbl.gov. Besides browsing, searching for various data of interest, and carrying out analytical tasks (see below), users can download the Annotators Database, which includes all of the annotated data elements and experiments as a sql dump file to perform their own analysis, as well as the Annotators Database Schema Description, and Alignments of Binding Site through the ‘Download’ page.

#### Data navigation

We developed a new navigation interface to easily select a set of experimental records based on six categories (classifications) covering different aspects of the database.

**Figure 1:**
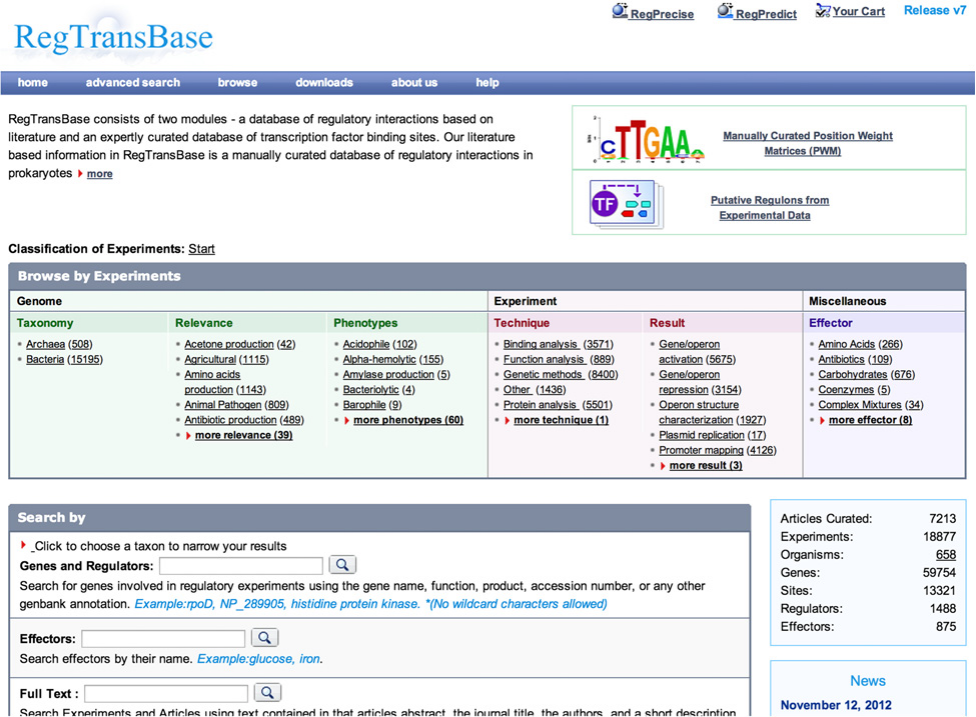
**Home page of RegTransBase.** Data navigation panel with its major classifications in the middle of the page.

Three categories (classifications) describe genomes that were studied in relevant experiments (Figure 1).

The ‘Taxonomy’ category is based on the NCBI Taxonomy [29] and describes phylogenetic relationships. A user can choose a taxon of interest starting from the super kingdom level (Bacteria or Archaea) and move down to the species level. The ‘Relevance’ category refers to the attributes of genome projects that provide information about the wide area of research a particular genome is a part of, such as Antibiotic production, Agricultural, etc. [30]. The ‘Phenotypes’ category includes attributes that describe phenotypic properties of the organisms [30].

Two categories refer to experimental methodology and the goals of experiments. The ‘Experiment techniques’ classification uses a controlled vocabulary of methods used in experiments. This classification has a two-level structure with the upper level containing method categories (i.e. protein analysis, RNA analysis) and lower level containing individual techniques such as Western blotting, DNAase footprinting etc. The ‘Experiment result’ classification describes what the experiment resulted in (i.e. promoter mapping, regulatory site mapping, gene/operon repression).

The ‘Effector’ classification uses a tree-like hierarchy of effectors where classes of the hierarchy are mainly based on the Chemicals and Drugs Category of MESH [31].

User can browse all categories in the database by choosing a term in one classification and then narrowing a result by choosing terms in other classifications as additional filters. At any time, the user can click on the number beside the classification to get articles fitting all criteria currently selected.

**Figure 2:**
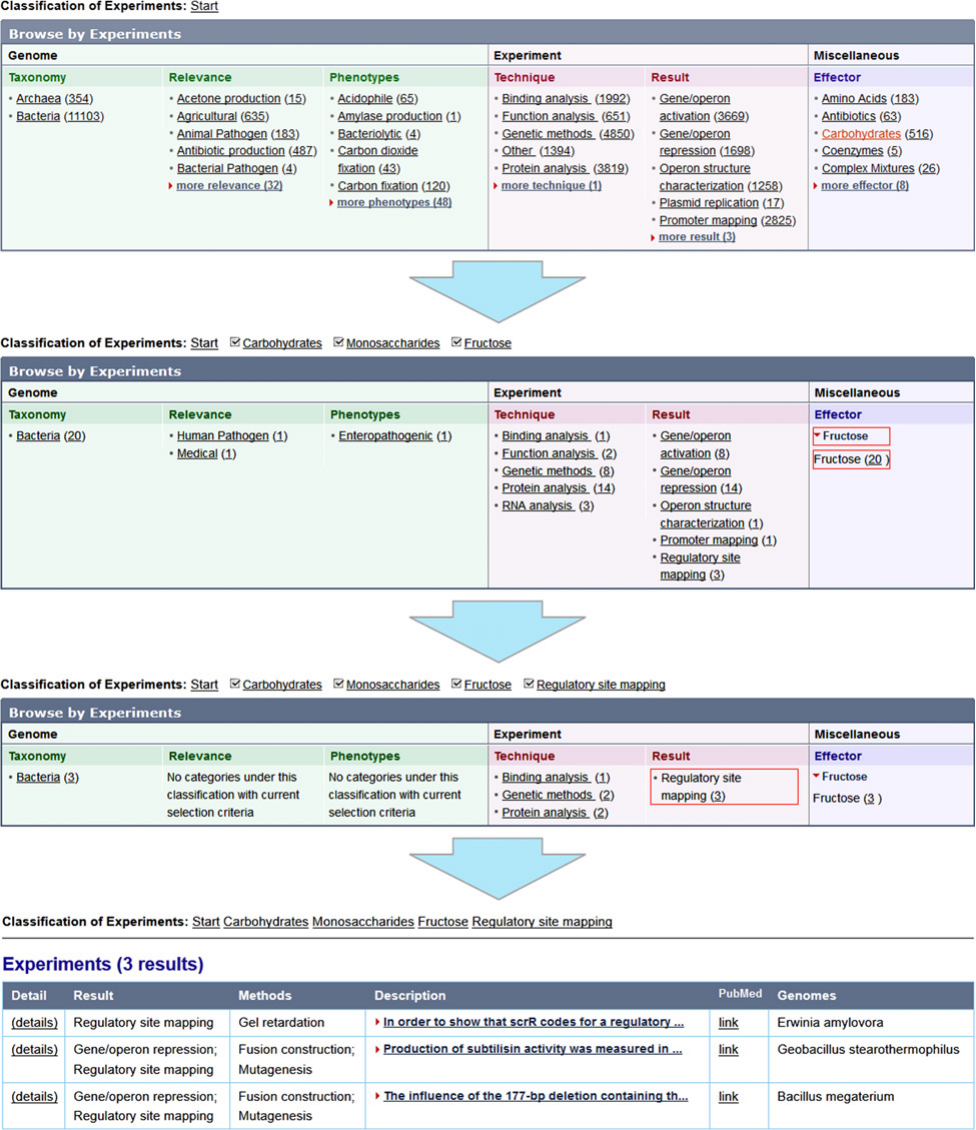
Step-by-step data navigation in search for the experiments where cis-elements are involved in the fructose-dependent regulation.

For example, we want to know if there is any data on experiments with cis-elements that are involved in fructose-dependent regulation. By using the ‘Effectors’ classification in three steps: ‘Carbohydrates’ -> ‘Monosaccharides’ -> ‘Fructose’ we find a list of 20 experiments (Figure 2).

A subsequent choosing of the ‘Regulatory site mapping’ term in the ‘Result’ classification produces a list of 3 experiments where cis-elements involved in fructose-dependent regulation were studied.

#### Search methods

RegTransBase provides a user with a broad range of search options such as search by Gene name, effector name, or a full text search of an abstract. Search for genes involved in regulatory experiments can be done using the gene name, function, product, accession number, or any other GenBank annotation. Searching for effectors by their name extracts the information on regulator, experiment, and genome with all associated links. Full text search allows for running complex queries against the abstracts and experiment descriptions such as *‘+mga +promoter’.*

#### Putative regulons from experimental data

Identification of transcription factor binding motifs is an important step in the computational reconstruction of regulatory elements. The ‘Putative Regulons’ section of RegTransBase provides sets of upstream sequences of target genes for each regulon. These sets can be used for the identification of conserved DNA motifs that may bind transcriptional regulators.

Use Case 1: use of Putative Regulon for the search of a TF binding motif

1. Find genome of interest on the Putative Regulons page.

2. Find regulon of interest based on the regulator name.

3. Get a set of upstream sequences by clicking the ‘Download’ link in the ‘Upstream sequences column of regulons table.

4. Start RegPredict [23], select genomes of interest.

5. Open ‘Discover Profiles’, paste upstream sequences (at least three sequences).

6. Select profile parameters (palindrome recommended), start search.

7. Select profile with highest informational content and run search for sites in selected genomes.

This scheme was successfully tested for the TnrA regulon from *B. subtilis*.

#### Manually curated position weight matrices (PWM)

**Figure 3:**
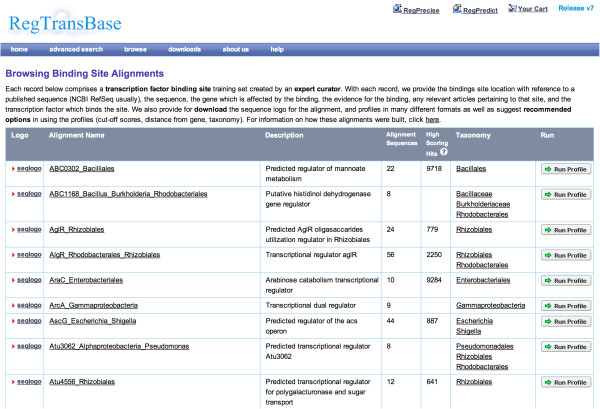
Access to the RegTransBase PWMs and browsing capabilities.

Positional weight matrices from RegTransBase collections can be used for computational prediction of TFBSs using RegPredict [23] or other software for PWM-based TFBS search. Figure 3 shows an access page to the RegTransBase PWMs and the associated data. A user selects a PWM of interest from the list and opens a webpage with PWM description. PWMs are available for download in different formats including a binding site alignment in FASTA format, matrices in MAST and Transfac formats and as a frequency matrix.

Use Case 2: use of manually curated PWM for computational reconstruction of a regulon

1. Open a list of the binding site alignments (http://regtransbase.lbl.gov/cgi-bin/regtransbase?page=alignment_browse).

2. Find a regulator of interest (for example, ABC0302).

3. Open the page with the ABC0302 binding sites alignment (http://regtransbase.lbl.gov/cgi-bin/regtransbase?page=show_alignment&amp;matrix_id=95).

4. Download an alignment in FASTA format (First option in Download section at the bottom of the page).

5. Go to the RegPredict website (http://regpredict.lbl.gov/).

6. Start RegPredict (click ‘Start Application’)

7. Click ‘Select genomes’.

8. Find recommended taxonomical group (Bacillales - see the ‘Recommended options’ section on ABC0302 page in RegTransBase) and add all genomes from that group (or as many genomes as possible).

9. Click ‘Run Profile’.

10. Select the ‘Sequences’ tab and paste your alignment of binding sites in the FASTA format.

11. Click ‘Generate profile’.

12. Set search parameters ‘Position from’ and ‘Position to’ (see ‘Recommended options’ section on ABC0302 page in RegTransBase).

13. Click ‘Run’.

## Conclusions

RegTransBase, a user-friendly open-access database, provides biologists involved in the investigation of microbial regulation and systems biology with convenient access to experimental data collected in thousands of original studies. It allows a user to interact with a valuable collection of manually curated data on a range of experiments related to the transcriptional regulation of bacteria. These data, with associated analytical tools, provide a valuable resource to assist in investigation of gene functions in the constantly growing number of available genome assemblies. RegTransBase collection of PWMs is currently used by various tools for TF binding prediction and motif comparison (for example, MEME-ChIP [32] and TOMTOM [33] from MEME Suite, FITBAR [34], ISGA [35], STAMP [13]. MicrobesOnline, an integrated portal for comparative and functional genomics [36], is cross-linked with RegTransBase.

As regulon inference is of significant importance for deciphering the regulation of biological processes, we believe that a current improved and expanded version of RegTransBase is a useful tool for the research community.

## Availability and requirements

RegTransBase is available at http://regtransbase.lbl.gov.

## Competing interests

The authors declare that they have no competing interests.

## Authors’ contributions

MJC worked on the database and interface design, general data organization and access; PSN participated in the database and interface design and construction, and lead the putative regulon collection and RegPredict access projects; AEK was responsible for data collection and manual curation; DAR proposed several critical directions of the project and actively participated in discussions; APA was involved with the MicrobesOnline integration and general discussions; MSG conceived and performed general coordination of the project. ID supervised the project and was involved with all aspects of database design, construction and implementation. MJC, AEK and ID wrote the manuscript. All authors read and approved the final manuscript.
